# Integrated Transcriptomics and Metabolomics Analysis Reveal the Regulatory Mechanisms Underlying Sodium Butyrate-Induced Carotenoid Biosynthesis in *Rhodotorula glutinis*

**DOI:** 10.3390/jof10050320

**Published:** 2024-04-27

**Authors:** Xingyu Huang, Jingdie Fan, Caina Guo, Yuan Chen, Jingwen Qiu, Qi Zhang

**Affiliations:** Faculty of Life Science and Technology, Kunming University of Science and Technology, Kunming 650500, China; xingyuh1997@foxmail.com (X.H.); fanl001@outlook.com (J.F.); 18468150409@163.com (C.G.); cheny@kust.edu.cn (Y.C.)

**Keywords:** *Rhodotorula glutinis*, carotenoids, sodium butyrate, transcriptome, metabolome

## Abstract

Sodium butyrate (SB) is a histone deacetylase inhibitor that can induce changes in gene expression and secondary metabolite titers by inhibiting histone deacetylation. Our preliminary analysis also indicated that SB significantly enhanced the biosynthesis of carotenoids in the *Rhodotorula glutinis* strain YM25079, although the underlying regulatory mechanisms remained unclear. Based on an integrated analysis of transcriptomics and metabolomics, this study revealed changes in cell membrane stability, DNA and protein methylation levels, amino acid metabolism, and oxidative stress in the strain YM25079 under SB exposure. Among them, the upregulation of oxidative stress may be a contributing factor for the increase in carotenoid biosynthesis, subsequently enhancing the strain resistance to oxidative stress and maintaining the membrane fluidity and function for normal cell growth. To summarize, our results showed that SB promoted carotenoid synthesis in the *Rhodotorula glutinis* strain YM25079 and increased the levels of the key metabolites and regulators involved in the stress response of yeast cells. Additionally, epigenetic modifiers were applied to produce fungal carotenoid, providing a novel and promising strategy for the biosynthesis of yeast-based carotenoids.

## 1. Introduction

Carotenoids comprise an essential group of natural lipophilic pigments. They consist of eight isoprene units connected in a head-to-tail manner and feature multiple conjugated double bonds, giving carotenoids their unique chemical structure [1]. As a result, carotenoids possess antioxidant activity. They can neutralize free radicals by donating electrons [2], aiding cells in combating oxidative stress, reducing the extent of lipid peroxidation, and maintaining the integrity of the cell membrane [3]. As food additives, carotenoids not only make food appear colorful [4] but also offer various health benefits to humans, such as providing vitamin A, strengthening the immune system, and reducing the risk of cardiovascular diseases and certain types of cancer [5]. Therefore, carotenoids have various important applications in multiple fields [6,7], and thus have high commercial value in the global market [8].

The industrial production of carotenoids mainly includes natural extraction and microbial fermentation. Microbial fermentation offers better control, higher efficiency, and better sustainability than natural extraction, making it a more prevalent and viable method for industrial production. Researchers have developed various approaches and strategies to increase the conversion efficiency of raw materials and maximize the production of carotenoids. These include fermentation control strategies, metabolic and genetic engineering approaches, and the application of adaptive laboratory evolution (ALE) to obtain more efficient fermentation strains [9,10,11]. Although these strategies have been used for decades to produce lipids and pigments in oleaginous microorganisms, more effective strategies need to be developed to enhance the yield of carotenoids and other products.

Fungal secondary metabolites are highly complex and diverse. However, under traditional laboratory culture conditions, many gene clusters responsible for controlling the expression of secondary metabolites are often silent [12,13]. Chemical epigenetic modifiers can modulate the structure and accessibility of chromatin, thus influencing the transcriptional activity of these gene clusters. Several studies have suggested that chemical epigenetic manipulation can trigger potential biosynthetic pathways and increase the expression of natural metabolites without altering genes or causing the hereditable manipulation of organisms [14,15,16]. Sodium butyrate (SB), a type of histone deacetylase inhibitor that affects chromatin structure and gene expression by blocking the activity of histone deacetylase, has been widely used in studies that investigate methods of promoting fungal secondary metabolite synthesis. For example, 9 µM SB was found to significantly increase the production of cytochalasin E, patulin, and pseurotin A when compared to the control in a suspension culture of *Aspergillus clavaus* [17]. Treatment with 10 mM SB in marine-derived *Penicillium brevicompactum* enhanced the production of anthranilic acid and ergosterol peroxide [18]. Similarly, treatment of the coral-derived fungus *Trichoderma harzianum* XS-20090075 with 10 µM SB induced an increase in the production of terpenoids, resulting in the identification of three new terpenoids and 11 known sesquiterpenoids [19]. *Rhodotorula glutinis* is one of the most extensively studied oleaginous yeasts. When grown at suitable temperatures, it can produce fatty acids, carotenoids, extracellular polysaccharides, and other compounds [20]. Although several studies have evaluated the effects of different culture conditions on carotenoid and lipid production in *Rhodotorula glutinis* [21,22,23], the specific effects and regulatory mechanisms of SB treatment have not been investigated.

In this study, we used the *Rhodotorula glutinis* strain YM25079, isolated from Lugu Lake in Lijiang City, Yunnan Province, China, to investigate the effect of treatment with SB on carotenoid biosynthesis and the underlying regulatory mechanisms. The mechanism of action was determined through integrated transcriptomic and metabolomic analyses. These findings contributed to a deeper understanding of the molecular and metabolic basis of carotenoid biosynthesis in YM25079 and provided insights into feasible fermentation processes and the molecular basis for increasing carotenoid production.

## 2. Materials and Methods

### 2.1. Strains and Cultivation Condition

The *Rhodotorula glutinis* strain YM25079 was isolated from Lugu Lake in Lijiang City, Yunnan Province, China, and the species was identified following the methods outlined in the referenced report [24]. YM25079 was preserved in a YPD liquid medium (yeast extract: 10 g/L, peptone: 20 g/L, and glucose: 20 g/L) supplemented with 35% glycerol and stored in a freezer at −80 °C for future use. Before conducting experiments, the strain was inoculated in a 250 mL conical flask containing 50 mL of YPD liquid medium. The pre-culture was incubated in a YRZ 201 shaking incubator (Shanghai Xilei Biotech Co., Ltd., Shanghai, China) at 160 rpm and 15 °C for 24 h. Subsequently, the pre-cultured YM25079 strain (inoculum: 0.5 mL) was inoculated in regular YPD liquid medium (control group) and YPD liquid medium supplemented with SB at different concentrations (experimental group). The cultures were then incubated at 160 rpm and 15 °C for 168 h in the incubator. All experiments were conducted in triplicate.

### 2.2. Analysis of Strain Growth and Intracellular ROS Levels

The growth curve of YM25079 was generated using the Prism 8 software and based on OD600 measurements taken at intervals of 24 h. The level of reactive oxygen species (ROS) in the culture medium was assessed after 96 h using a ROS assay kit (S0033S, Shanghai Biyuntian Biotechnology Co., Ltd., Shanghai, China). The fermentation broth was collected every 24 h, centrifuged, washed, and dried until a constant weight was achieved to determine the dry biomass. The biomass concentration (g/L) was calculated by dividing the dry biomass weight (g) by the volume of the fermentation broth (L). The dry biomass obtained was pulverized into a powder and stored at −80 °C for subsequent experiments.

### 2.3. Measurement of Total Lipid, Carotenoid, and Fatty Acid Composition

The determination of lipid content followed a previously reported method [25]. A 4 mol/L hydrochloric acid aqueous solution was added to the dry yeast powder and was heated in a boiling water bath for 3 min. Cooling to room temperature, the sample was centrifuged to collect the bottom chloroform layer after adding a mixture of chloroform and methanol (2:1, volume ratio). In addition, 0.1% (*w*/*v*) NaCl solution was added to the chloroform layer, followed by a repetition of the centrifugation process. Finally, the bottom chloroform layer was evaporated to a constant weight for the estimation of total lipid content using the weight method.

Carotenoids were extracted as described previously with slight modifications [26,27]. In summary, the yeast powder samples were further ground in a quartz mortar and dissolved in acetone. Subsequently, the upper organic phase was collected, and the lower phase was extracted twice. Total carotenoid concentration was determined by measuring the absorbance value at 450 nm (OD450) of the acetone extracts with a UV–Vis spectrophotometer (UV-1800PC, MAPADA, Shanghai, China), expressed as mg/g dry cell weight (DCW). The content of carotenoid was calculated using the formula “X = 1000 EV/AW”, where X is the total amount of carotenoid (mg/g DCW), E is OD450, V is the total volume of acetone extracts (mL), W is the weight of dried powder sample (g), and A is an average extinction coefficient (2500) for carotenoid.

### 2.4. RNA Sequencing (RNA-seq) and Data Analysis

Total RNA from the YM25079 cells was extracted following the instructions provided with the TRIzol^®^ Reagent Kit (Thermo Fisher Scientific, Waltham, MA, USA). After assessing purity, concentration, and integrity, the transcriptional library was constructed using the Illumina TruSeqTM RNA Sample Prep Kit (Illumina, San Diego, CA, USA), and sequencing was performed using the Illumina NovaSeq 6000 sequencer (Illumina, San Diego, CA, USA).

After the quality of the raw data was assessed, adapter sequences and low-quality reads were removed using the fastp software (version 0.23.4). Following this, high-quality clean reads were mapped to the reference genome using HISAT2 (version 2.1.0) with default parameters. The RSEM software (version 1.3.3) was used to quantify the level of expression of genes and transcripts. The DESeq2 software (version 1.42.0) was used to analyze read counts and determine the differences in the expression of genes between samples and groups. Statistical and enrichment analyses of differentially expressed genes were conducted using the GOATOOLS library and the KOBAS software (version 3.0.3).

### 2.5. Real-Time Quantitative PCR Analysis

To assess the quality of the transcriptome sequencing data (BioProject ID: PRJNA1091310), a subset of differentially expressed genes was used for RT-qPCR analysis. The RT-qPCR primers were designed using the NCBI Primer-BLAST (https://www.ncbi.nlm.nih.gov/tools/primer-blast/, accessed on 15 November 2023), while the primers were synthesized by Shanghai Shenggong Bioengineering Co., Ltd. (Shanghai, China). The RNA, which was stored at −80 °C, was reverse transcribed using the HiScript II 1st Strand cDNA Synthesis Kit (+gDNA wiper) (R212-02, Vazyme, Nanjing, China). The resulting cDNA served as a template for real-time quantitative PCR analysis to compare the differences in gene expression. The RT-qPCR validation primers are listed in Table S1.

### 2.6. Metabolomics and Data Analysis

The YM25079 strain was harvested after 120 h of fermentation. Yeast cells were centrifuged to remove the supernatant and subsequently stored at −80 °C. The samples were transported under cold chain conditions to Shanghai Biotree Biomedical Technology Co., Ltd. (Shanghai, China) for metabolite extraction and liquid chromatography−tandem mass spectrometry (LC-MS/MS).

The raw data obtained post-sequencing were converted to mzXML files using the MSConvert tool in the Proteowizard software package (version 3.0.21229). Following this, the detection, filtering, and alignment of the peaks were conducted using the RxCMS software package (version 3.12.0). The metabolites were identified using public databases such as the Kyoto Encyclopedia of Genes and Genomes (KEGG), LipidMaps, MassBank, mzCloud, etc. The MetaboAnalyst software (version 5.0) was used for pathway enrichment and topological analysis of the selected differentially expressed metabolites. Subsequently, KEGG Mapper, a visualization tool, was used to examine the differential metabolites and pathway diagrams enriched by the identified metabolites.

## 3. Results

### 3.1. Effects of Different Concentrations of SB on the Growth and Carotenoid Accumulation in the YM25079 Strain

**Figure 1:**
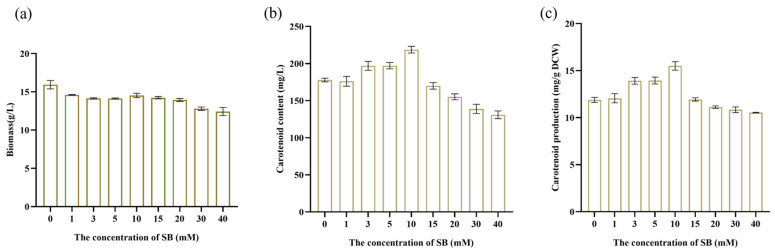
Effects of treatment with SB on the total biomass, carotenoid content, and carotenoid production by YM25079. (**a**) Total biomass, (**b**) carotenoid content, (**c**) carotenoid production. The data are presented as the mean ± standard deviation of triplicates.

In this study, the differences in total biomass, carotenoid content, and production of YM25079 were evaluated after treatment with various concentrations of SB. The results showed that the biomass was lower after treatment with all concentrations of SB (1 mM, 3 mM, 5 mM, 10 mM, 15 mM, 20 mM, 30 mM, and 40 mM) compared to that in the control (0 mM SB), and it also decreased significantly after treatment with 40 mM SB (Figure 1a). The carotenoid content of YM25079 increased after treatment with lower concentrations of SB (3 mM, 5 mM, and 10 mM) after 120 h of cultivation in comparison to the carotenoid content in the control group. It increased significantly after treatment with 10 mM SB; however, no significant change in the carotenoid content was observed after treatment with 1 mM SB (Figure 1b). The carotenoid content decreased after treatment with high concentrations of SB (15 mM, 20 mM, 30 mM, and 40 mM). Like carotenoid content, carotenoid production was the highest and lowest after treatment with 10 mM and 40 mM SB, respectively (Figure 1c). Based on these results, 10 mM SB was selected as the optimal concentration to increase the biosynthesis of carotenoids in YM25079, and this concentration of SB was used for further analyses.

### 3.2. Effects of 10 mM SB on the Growth, Lipid, Carotenoid, and ROS in the YM25079 Strain

**Figure 2:**
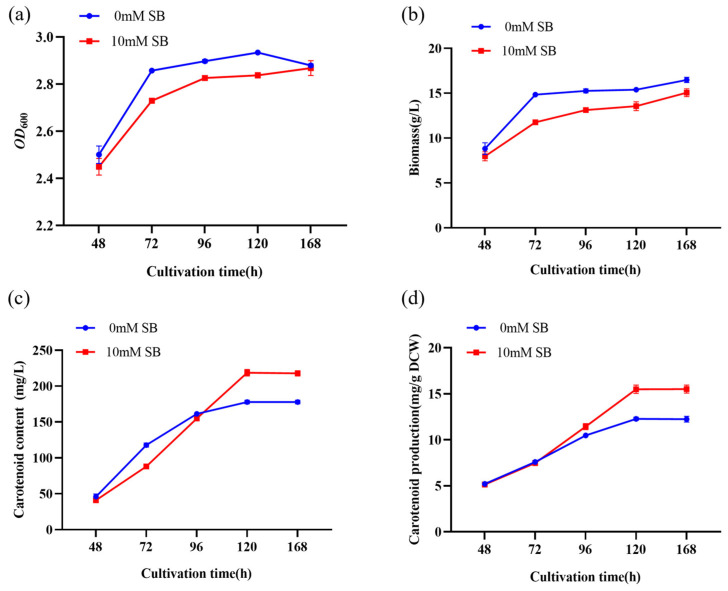
Effects of 10 mM SB on the growth curve, biomass, carotenoid content, carotenoid production of YM25079. (**a**) Growth curve, (**b**) total biomass, (**c**) carotenoid content, and (**d**) carotenoid production. The data are presented as the mean ± standard deviation of triplicates.

**Figure 3:**
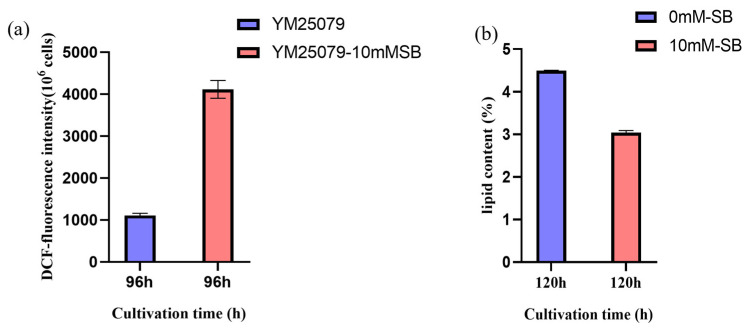
(**a**) Effects of 10 mM SB on ROS at 96 h. (**b**) The lipid content after the addition of SB at 120 h. The data are presented as the mean ± standard deviation of triplicates.

To better understand the effect of 10 mM SB on the physiological parameters of the YM25079 strain, we cultured YM25079 with 10 mM SB for 168 h, with sampling being performed every 24 h after the initial 48 h. The results showed that the growth rate of YM25079 in the experimental group (Y79_96SB) decreased slightly compared to the growth rate in the control group (Y79_96), starting from 48 h, but it was similar at 168 h (Figure 2a). However, the biomass in the experimental group was consistently lower than that in the control group (Figure 2b). The content and production of carotenoids were higher in the experimental group than in the control group after 96 h, but they stabilized around 120 h (Figure 2c,d) and exhibited higher ROS levels after 96 h (Figure 3a). Furthermore, the lipid content in the experimental group was decreased after the addition of SB (Figure 3b).

### 3.3. Transcriptome Analysis of the YM25079 Strain in Response to SB Treatment

To evaluate the transcriptional response of the YM25079 strain to SB stress, a transcriptome analysis was performed on two groups of YM25079 strains after 96 h of cultivation (n = 6 samples). In total, 260 million raw reads were generated, and, after filtering, 4.4 million clean reads were retained for each sample on average (Table S2). After ribosomal RNA was removed, 97.72–98.28% of clean reads were successfully mapped to the reference genome of the YM25079 strain using the TopHat2 software (version 2.1.1) (Table S3). These results confirmed that the assembled transcripts were of high quality, thus ensuring the accuracy of subsequent analyses.

### 3.4. Identification of DEGs in Response to SB Treatment

**Figure 4:**
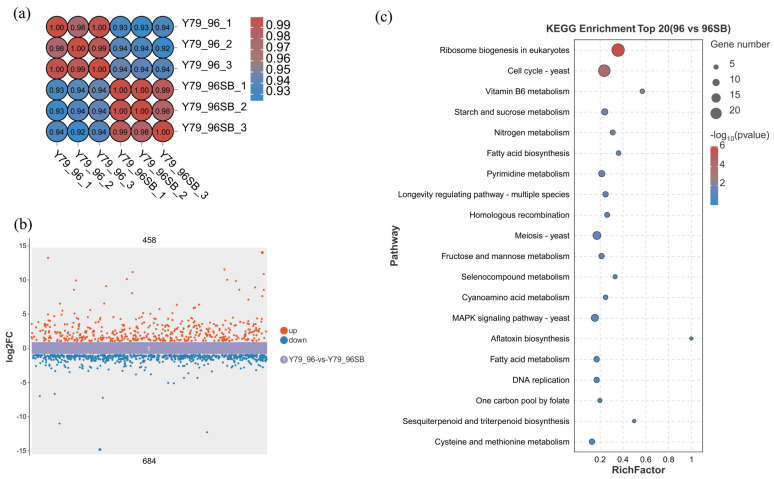
Comparative transcriptome analysis of YM25079 after treatment with 10 mM SB. (**a**) Correlation heatmap analysis. (**b**) DEGs scatter plot. (**c**) Top 20 KEGG pathways enriched by DEGs. Note: Y79 is the abbreviation of YM25079, 96 represents the culture time, and SB represents bu-tyrate treatment.

The correlation heatmap showed a significant difference between Y79_96 and Y79_96SB, which indicated that treatment with 10 mM SB strongly affected the level of gene expression of YM25079 (Figure 4a). Using the DESeq2 software (version 1.42.0) with a threshold of FDR < 0.05 and absolute log2 (fold change with FPKM) ≥ 1, we identified 1142 differentially expressed genes. Among them, 458 (40.11%) genes were upregulated and 684 (59.89%) genes were downregulated (Figure 4b). To determine the molecular changes in YM25079 after exposure to SB, we conducted KEGG and gene ontology (GO) enrichment analyses. The results of the KEGG analysis revealed that 91 pathways were enriched, and the top 20 pathways included Genetic Information Processing (KO03008 and KO03030), Cell Cycle (KO04111), Metabolism of Cofactors and Vitamins (KO00750 and KO00670), Carbohydrate Metabolism (KO00500 and KO00051), Nitrogen Metabolism (KO00910), Lipid Metabolism (KO00061 and KO01212), Amino Acid Metabolism (KO00450, KO00460, and KO00270), Signal Transduction (KO04011), Metabolism of Terpenoids and Polyketides (KO00909), etc. (Figure 4c).

For the comprehensive functional characterization of total DEGs, GO analysis was conducted. The DEGs could be classified into 44 subcategories across 3 main categories, with 24 subcategories in Biological Process, 17 in Molecular Function, and three in Cellular Component. In the Biological Process category, the top three enriched pathways were Cellular Process, Metabolic Process, and Biological Regulation. In the Molecular Function and Cellular Component categories, the top three enriched pathways were Binding, Catalytic Activity and Transporter Activity; Cellular Anatomical entity, Protein-Containing Complex, and Virion Component (Figure S1). Additionally, in the Biological Process category, the most significantly enriched pathways for DEGs were rRNA metabolic processes (GO:0016072), ribosomal small subunit biogenesis (GO:0042274), and rRNA processing (GO:0006364). In Molecular Function, the most significant enrichments were associated with snoRNA binding (GO:0030515), U3 snoRNA binding (GO:0034511), and solute: proton symporter activity (GO:0015295). In Cellular Component, the most significant enrichments were associated with preribosome (GO:0030684), nucleolus (GO:0005730), and 90S preribosome (GO:0030686) (Table S4). These results suggested that gene expression regulation, membrane transport, and organic metabolic processes were most active after treatment with 10 mM SB.

To confirm the accuracy of the transcriptome data under investigation, a random selection of 12 DEGs was used to perform a quantitative reverse transcription−polymerase chain reaction (qRT−PCR) for quantitative analysis. All DEGs showed a generally consistent expression pattern between the qRT−PCR results and RNA-seq data (Figure S2), which confirmed that the RNA-seq dataset is suitable for further analysis.

### 3.5. Non-Targeted Metabolomic Analysis of Differentially Accumulated Metabolites

**Figure 5:**
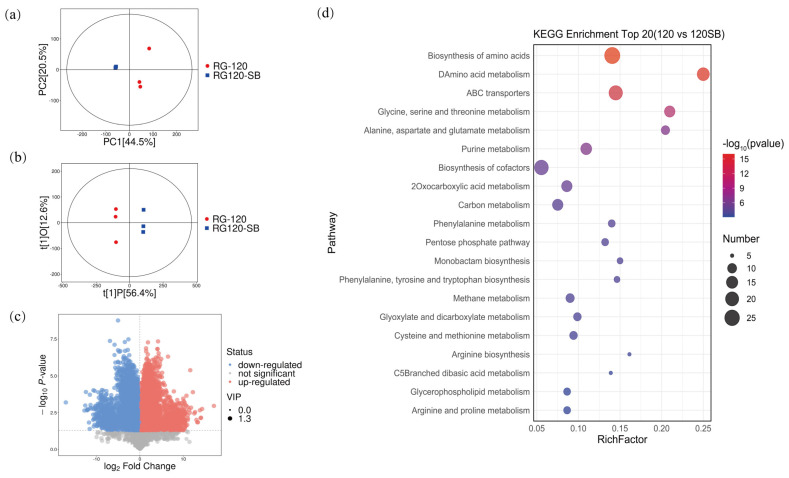
General overview of differentially accumulated metabolites (DAMs) in YM25079 after treatment with 10 mM SB. (**a**) PCA score scatter plots. (**b**) OPLS−DA scatter diagram. (**c**) Volcano plots of the DAMs. (**d**) The top 20 KEGG enrichment terms for RG120 and RG120SB.

To further elucidate the changes in the metabolites induced by SB treatment in YM25079, non-targeted metabolomic profiling was performed by conducting liquid chromatography−tandem mass spectrometry (LC−MS/MS) on the samples from both groups cultured for 120 h (RG-120 and RG120-SB). The results of principal component analysis (PCA) revealed significant differences between the experimental and control groups (Figure 5a). Additionally, the orthogonal partial least squares discriminant analysis (OPLS-DA) indicated that the results were highly reproducible, enabling further analysis of differential metabolites (Figure 5b).

In total, 638 metabolites were co-detected in the total ion chromatogram (TIC); among these metabolites, organic acids and derivatives (30.50%), organ heterocyclic compounds (16.13%), and lipids and lipid-like molecules (15.84%) were the most abundant (Figure S3). Furthermore, 341 metabolites were identified as differential metabolites, including 196 upregulated and 145 downregulated (Figure 5c). The results of the KEGG enrichment analysis revealed that the differential metabolites were enriched in 72 metabolic pathways (Table S5). The top 20 pathways included amino acid biosynthesis (sce01230), amino acid metabolism (sce00470, sce00260, sce00250, sce00360, sce00400, sce00270, sce00220, and sce00330), ABC transporters (sce02010), carbon metabolism (sce01200), and glycerophospholipid metabolism (sce00564) among others (Figure 5d).

After treatment with 10 mM SB, YM25079 accumulated a large amount of organic acids. Among them, the levels of Phenylpyruvic acid (log2 FC = 17.03), L-Pipecolic acid (log2 FC = 13.99), and 3-hydroxytetradecanoic acid (log2 FC = 4.97) increased the most. In contrast, the levels of gamma-Aminobutyric acid (log2 FC = −5.03), 4-Deoxyerythronic acid (log2 FC = −5.02), and Argininosuccinic acid (log2 FC = −3.54) decreased the most. The abundance of most amino acids, such as L-arginine (log2 FC = 3.04), L-methionine (log2 FC = 2.00), S-adenosylhomocysteine (log2 FC = 1.53), L-histidine (log2 FC = 1.23), L-phenylalanine (log2 FC = 1.20), L-tryptophan (log2 FC = 1.19), L-leucine (log2 FC = 1.16), L-threonine (log2 FC = 1.14), and L-glutamic acid (log2 FC = 1.06), increased. However, the abundance of L-arginine (log2 FC = −1.39) and pyruvic acid (log2 FC = −0.29) decreased, suggesting that they might be consumed during the stress adaptation process (Table S6).

### 3.6. Integrative Analysis of Transcriptome and Metabolome

**Figure 6:**
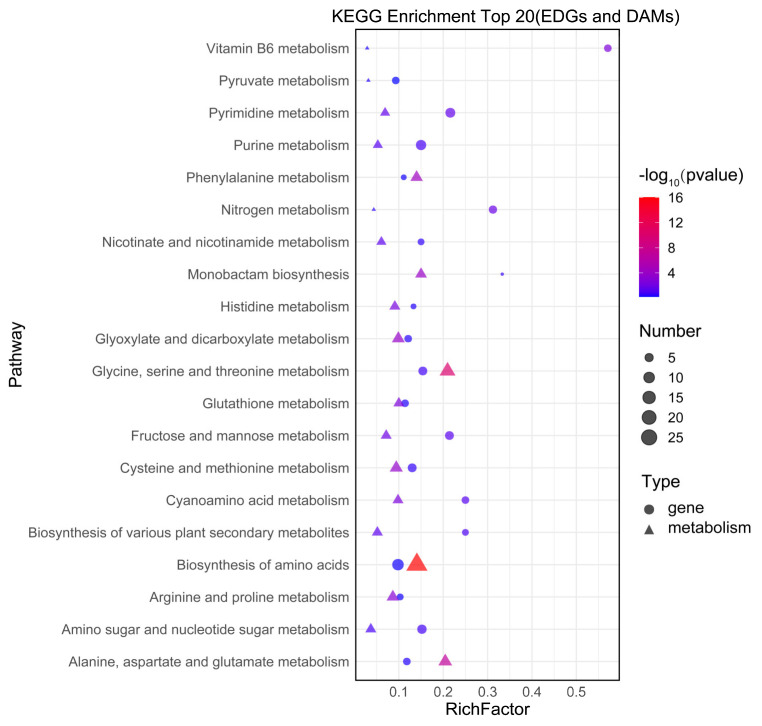
Integrated analysis of the top 20 KEGG pathways enriched in DEGs and DAMs. The X-axis indicates the enrichment score of the DEGs and DAMs. The *p*-value is indicated using a color scale; the size of the dots and triangles indicate the number of DEGs and DAMs mapped in each pathway, respectively.

To elucidate the mechanism underlying the regulatory effect of 10 mM SB in promoting carotenoid biosynthesis, a comprehensive transcriptomic and metabolomic analysis was conducted. The results of the KEGG analysis revealed that DEGs and differentially accumulated metabolites (DAMs) were enriched in 52 pathways (Table S7), including 14 pathways related to amino acid metabolism, 10 pathways related to carbohydrate metabolism, and eight pathways related to cofactor and vitamin metabolism. The most enriched pathways included the metabolism of vitamin B6, pyruvate, pyrimidine, purine, and phenylalanine (Figure 6). Additionally, pathways such as the metabolism of cysteine, methionine, alanine, aspartate, glutathione, proline, asparagine, and glutamate were well enriched, indicating that these metabolic pathways responded significantly to the SB added.

#### 3.6.1. SB Affects the Antioxidant System of the YM25079 Strain by Regulating Gene Expression

The antioxidant system is a collective term for a series of defense mechanisms in living organisms that can counteract oxidative stress and maintain the redox balance within cells. The primary components of the antioxidant system include various enzymes, proteins, and small molecules that work together to alleviate or prevent cell damage caused by oxidative stress. These components encompass enzymes such as superoxide dismutase (SOD), catalase, the glutathione peroxidase system, glutathione reductase, and non-enzymatic antioxidant molecules.

**Figure 7:**
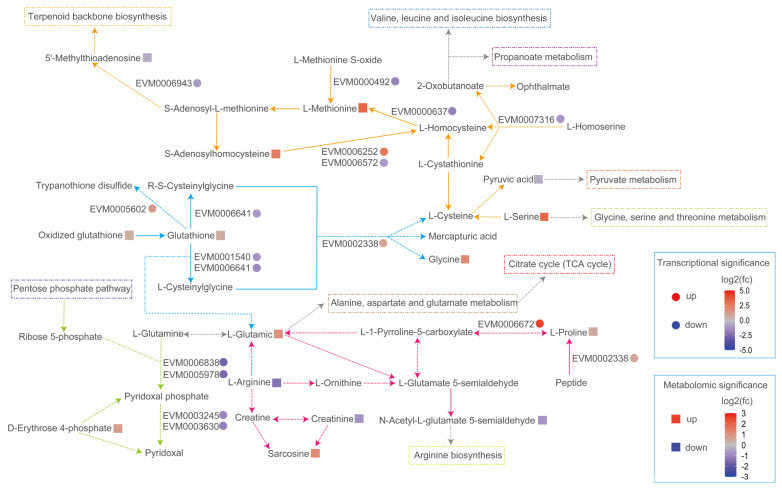
Overview of selected antioxidant-related metabolic pathways in response to treatment with SB. The colors of ellipses and rectangles indicate significance, which is presented on a color scale. Arrows of different colors represent different metabolic pathways (Blue—glutathione metabolism, yellow—methionine and cysteine metabolism, pink—arginine and proline metabolism, green—vitamin B6 metabolism, gray—other related pathways). Dashed arrows indicate the simplified pathways.

When exposed to 10 mM SB, ROS levels increased in YM25079 (Figure 3a), which was associated with the effect on the antioxidant system of the strain. There were five DEGs and four DAMs, six DEGs and five DAMs, and two DEGs and five DAMs associated with glutathione metabolism, cysteine and methionine metabolism, and arginine and proline metabolism, respectively (Figure 7). The metabolites that accumulated significantly included L-methionine, S-Adenosylhomocysteine, L-glutamic acid, and L-serine, whereas the levels of L-arginine and pyruvic acid decreased. Simultaneously, the transcriptional levels of many associated genes were downregulated, such as Gamma-glutamyltransferase/gamma-glutamyl peptidase (EVM0006641, log2 FC = −1.1), gamma-glutamylcysteine synthetase (EVM0001540, log2 FC = −1.2), L-methionine (R)-S-oxide reductase (EVM0000492, log2 FC = −1.8), homocysteine methyltransferase (EVM0000637, log2 FC = −1.8), etc. Additionally, in vitamin B6 metabolism, the transcriptional levels of pyridoxine kinase (EVM0003245, log2 FC = −1.1), pyridoxal 5’-phosphate synthase pdxS subunit (EVM0006838, log2 FC = −2.8), 5’-phosphate synthase pdxT subunit (EVM0005978, log2 FC = −2.6), and pyridoxal phosphate phosphatase PHOSPHO2 (EVM0003630, log2 FC = −1.2) were downregulated. Moreover, the transcriptional levels of genes related to hydrogen peroxide (EVM0001641, log2 FC = −3.8, EVM0001756, log2 FC = −1.3) were downregulated (Table S8). These results indicated that SB altered the level of gene expression in YM25079, affecting its antioxidant system.

#### 3.6.2. Fructose and Mannose Metabolism Mitigate Membrane Damage Induced by Exposure to SB

**Figure 8:**
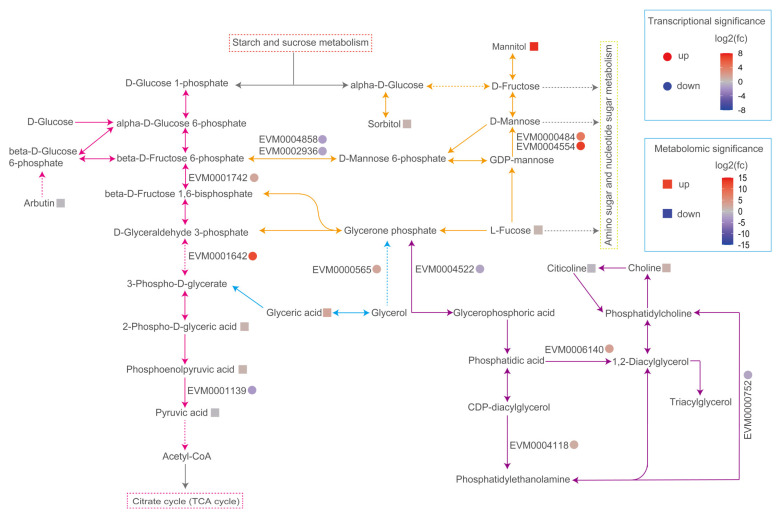
Overview of a part of the fructose and mannose metabolism in response to treatment with SB. The colors of ellipses and rectangles indicate significances, which are presented as a color scale. Arrows of different colors represent different metabolic pathways (Blue—glycerolipid metabolism, yellow—fructose and man-nose metabolism, pink—glycolysis/gluconeogenesis, purple—glycerophospholipid metabolism, gray—other related pathways). Dashed arrows indicate the simplified pathways.

Mannitol is a naturally occurring crystalline alditol that plays various roles in microbial organisms. In environments with high osmolarity, microorganisms may accumulate mannitol to maintain intracellular osmotic balance and prevent cell damage induced by osmotic stress. By regulating the fluidity and stability of the membrane, mannitol can protect the cell membrane of microorganisms, thus mitigating oxidative stress. After YM25079 was treated with SB, the fructose and mannose metabolism pathway exhibited enrichment with five DEGs and three DAMs. This enrichment included mannitol-endowed mannan endo-1,4-beta-mannosidase (EVM0000484, log2 FC = 4.2, EVM0004554, log2 FC = 7.1) and mannitol (log2 FC = 14.1) with remarkably high fold changes. Nine DEGs and seven DAMs associated with sugar glycolysis/gluconeogenesis, glycerol lipid metabolism, and glycerol phosphate metabolism were enriched. Metabolites such as glyceric acid, choline, and sorbitol showed significant accumulation, with most of the corresponding genes exhibiting transcriptional upregulation (Figure 8). Additionally, the level of expression of the key enzymes involved in chitin synthesis, including chitin synthase (EVM0000822, log2 FC = 2.6; EVM0005208, log2 FC = 1.0) and chitinase (EVM0000667, log2 FC = 1.9; EVM0003676, log2 FC = 1.9), increased. This upregulation contributed to chitin biosynthesis, providing structural support and protection for the cell wall. The accumulation of these metabolites and the upregulation of associated genes together decreased membrane damage caused by exposure to SB, which helped maintain the structural integrity and functionality of the cell membrane.

#### 3.6.3. YM25079 Counteracts ROS by Promoting Carotenoid Biosynthesis

**Figure 9:**
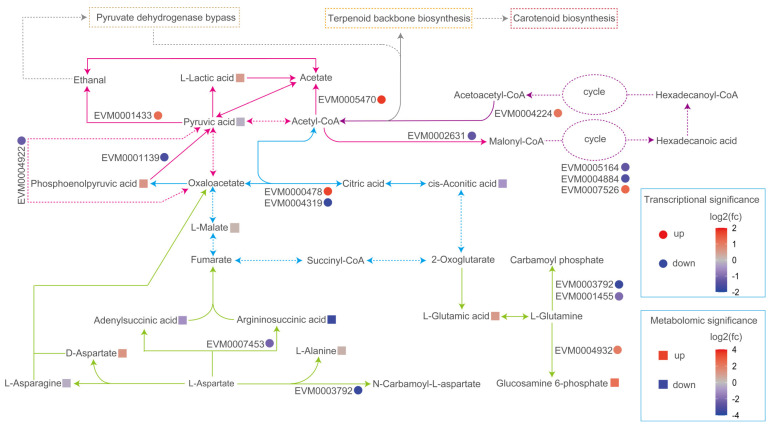
Overview of selected acetyl-CoA-related metabolic pathways in response to treatment with SB. The colors of ellipses and rectangles indicate significances, which are presented as a color scale. Arrows of different colors represent different metabolic pathways (Blue—TCA cycle, purple—fatty acid metabolism, pink—pyruvate metabolism, green—alanine, aspartate, and glutamate metabolism, gray—other related pathways). Dashed arrows indicate the simplified pathways.

Carotenoids in fungi not only make the colonies appear colorful but also protect them against oxidative stress caused by adverse environmental conditions. In yeast, carotenoids are synthesized using acetyl-CoA as a substrate and undergo three stages of synthesis. Acetyl-CoA, generated through the pyruvate dehydrogenase bypass (PDH bypass), is directly used in subsequent biosynthetic pathways, including fatty acids, terpenoids, derivative carotenoid, sterols, amino acids, and polyketides. After treatment with 10 mM SB, the metabolism of pyruvic acid, alanine, aspartic acid, and glutamic acid showed significant enrichment in YM25079. Five DEGs and three DAMs were found to be associated with pyruvic acid metabolism, and four DEGs and seven DAMs were associated with the metabolism of alanine, aspartic acid, and glutamic acid. Additionally, pathways related to acetyl-CoA, such as the TCA cycle and fatty acid metabolism, showed enrichment with six DEGs and two DAMs (Figure 9). Among these DAMs, glucosamine 6-phosphate (log2 FC = 2.3), D-aspartic acid (log2 FC = 1.3), aminoadipic acid (log2 FC = 1.2), argininosuccinic acid (log2 FC = −3.5), and adenylsuccinic acid (log2 FC = −1.1) exhibited significant changes. In contrast, the upregulation of pyruvate decarboxylase (EVM0001433, log2 FC = 1.1) promoted the conversion of pyruvic acid to acetaldehyde, reinforcing the pyruvate dehydrogenase bypass. The regulation of acetyl-CoA carboxylase (EVM0002631, log2 FC = −1.3), fatty acid synthase (EVM0004884, log2 FC = −1.5, EVM0005164, log2 FC = −1.3), and acetyl-CoA acyltransferase (EVM0004224, log2 FC = 1.1) led to a reduction in lipid content. Additionally, the upregulation of 4-coumarate--CoA ligase (EVM0005805, log2 FC = 1.5) and squalene monooxygenase (EVM0005546, log2 FC = 1.3) promoted the synthesis of terpenoids. These changes in metabolites and genes suggested that YM25079 may redirect more acetyl-CoA toward carotenoid biosynthesis.

## 4. Discussion

After the cultures of *Alternaria alternata* and *Penicillium expansum* treated with trichostatin A to activate the production of secondary metabolites were first reported in 2007, researchers have made significant progress in the use of chemical epigenetic modifiers to promote the formation of fungal products [14]. These modifiers can effectively stimulate the silencing or expression of fungal secondary metabolic pathways [15,16]. However, researchers have not investigated their application in carotenoid production. In this study, we found that treatment with 10 mM SB significantly increased the production of carotenoids in the YM25079 strain. Low concentrations of SB did not induce significant biological effects, which may be related to the dose dependence of epigenetic modifiers [28]. In contrast, high concentrations of SB can indeed affect the synthesis of metabolites, leading to a decrease in carotenoid production, which is consistent with some research findings [29,30]. Additionally, after SB treatment, the biomass of the YM25079 strain decreased (Figure 1a), which matched the conclusions of another study in which the inhibition of HDAC was found to suppress yeast growth [31]. In conclusion, these findings showed the feasibility of using the histone deacetylase inhibitor SB in carotenoid production in the YM25079 strain.

The correlation between carotenoid biosynthesis and fluctuations in intracellular oxidative stress levels has been confirmed [32,33,34]. Under oxidative stress, the production of oxidizing agents in intracellular and extracellular environments surpasses the inherent antioxidant capacity of the cell, leading to oxidative ROS age to biological molecules, such as lipids, proteins, and nucleic acids [35]. ROS can strongly influence oxidative stress; ROS include substances like superoxide anions (O2-), hydrogen peroxide (H2O2), hydroxyl radicals (·OH), etc. [36]. The levels of these substances are regulated to normal levels by enzymatic and non-enzymatic antioxidant systems [37]. Notably, β-carotene is the principal non-enzymatic antioxidant found in red yeast cells; in another study, we found a correlation between carotenoid biosynthesis and ROS accumulation in red yeast [38]. In this study, the experimental group (Y79_96SB) exhibited high ROS levels and increased carotenoid synthesis efficiency at 96 h (Figure 2c and Figure 3a). This finding suggested that the increase in carotenoid biosynthesis in the YM25079 strain might be a consequence of an increase in intracellular oxidative stress induced by SB treatment. However, no study has investigated the influence of SB on intracellular oxidative stress levels in yeast cells.

Based on our findings, we speculated that the high ROS levels observed in the experimental group (Y79_96SB) may be attributed to the disruption of the enzymatic antioxidant system. The enzymes involved in regulating the synthesis, degradation, and conversion of glutathione (an important intracellular antioxidant), including gamma-glutamyltranspeptidase/glutathione hydrolase (EVM0006641, log2 FC = −1.1) and glutathione-specific gamma-glutamylcyclotransferase (EVM0001540, log2 FC = −1.2), were inhibited. Additionally, a decrease in the ratio of reduced glutathione to oxidized glutathione (log2 FC = 0.37) disrupted the redox state of glutathione, affecting its antioxidant levels [39]. SAM is the main methyl donor and precursor of glutathione [40] that can stimulate the activity of glutathione S-transferase [41]. Under conditions where homocysteine methyltransferase (HCMT) is inhibited and PLP is deficient, homocysteine is primarily converted to SAH (log2 FC = 1.5) [42], which leads to the inhibition of SAM-dependent methylation reactions and effects on glutathione synthesis (Figure 7). When the activities of antioxidant enzymes are strongly inhibited or disrupted, the non-enzymatic antioxidant system can be activated as the second line of defense against ROS. This system acts as a supplementary antioxidant defense mechanism to counteract ROS and maintain the overall antioxidant capacity [32]. Studies have shown that inhibiting antioxidant enzyme activities enhances carotenoid biosynthesis in the microalga *Dactylococcus dissociatus*, particularly catalase [43]. In the YM25079 strain, catalase (EVM0001641, log2 FC = −3.8, EVM0001756, log2 FC = −1.3) is inhibited, which may positively influence the overproduction of carotenoid while weakening the antioxidant capacity of the strain. Under moderate levels of oxidative stress induced by unfavorable environmental conditions, red yeast can enhance the biosynthesis of carotenoid, thus increasing its tolerance to oxidative stress [44]. These findings suggested that 10 mM SB decreased the enzymatic antioxidant system of the YM25079 strain, leading to an increase in ROS levels and the subsequent enhancement of carotenoid biosynthesis.

High ROS levels disrupt the structure of lipid molecules on the cell membrane, thus altering the functionality, fluidity, and permeability of the membrane [45]. Cells maintain the integrity of the cell membrane under high ROS levels by adjusting the structure of the phospholipid bilayer, undergoing autophagy, and regulating antioxidant defense systems [46]. In this study, the content of ergosterol (log2 FC= −2.3) decreased significantly, which may be related to its property of protecting lipids on cell membranes from auto-oxidation [47]. However, this behavior also leads to changes in membrane fluidity and permeability. Carotenoids are the primary representatives of the non-enzymatic antioxidant system in red yeast, and they not only inhibit oxidative stress but also influence the physical properties of biological membranes through their unique chemical structure and physical properties [48]. Increasing the cellular carotenoid content can alter the fluidity and order of the membranes of living cells [49]. To maintain normal cell growth, YM25079 enhances the pathways associated with the synthesis of various metabolites, such as mannitol, glyceric acid (log2 FC = 2.3), choline (log2 FC = 1.5), etc. Mannitol is a polyol that maintains the structural stability of cells by regulating osmotic balance, clearing hydroxyl radicals, and reducing oxidative stress [50,51]. Glyceric acid is an oxidation product of glycerol that generates 3-phospho-D-glycerate and participates in the TCA cycle. Choline and its metabolites influence the normal growth and development of cells and protect the integrity and functionality of cell membranes through various pathways, such as regulating the composition, fluidity, antioxidant effects, and involvement of the membrane in cell signaling [52]. Additionally, betaine (log2 FC = 0.6) can act as a methyl donor, participating in methylation reactions in cells and facilitating the conversion of homocysteine [53]. Glycerol 2-dehydrogenase (EVM0000565, log2 FC = 1.5) and diacylglycerol diphosphate phosphatase/phosphatidate phosphatase (EVM0006140, log2 FC = 1.8) promote the conversion of glycerol to triacylglycerol, which provides energy to the cell and maintains cell membrane stability (Figure 8). Additionally, chitin synthase and chitinase facilitate chitin synthesis, providing structural support and protection for the cell wall [54]. These findings indicated that YM25079 responds to oxidative stress and the damaging effects of sodium butyrate on the cell membrane by modulating the metabolism of carotenoids, fructose, and mannose, along with their related pathways.

In yeast cells, acetyl-CoA serves as the substrate for carotenoid biosynthesis [55]. At an industrial scale, developing various strategies to enhance the pool of acetyl-CoA is an effective approach to promote the downstream production of carotenoids [56]. In our study, multiple pieces of evidence suggest that YM25079 synthesizes carotenoids to increase tolerance to ROS. An increase in the expression of pyruvate decarboxylase promoted the pyruvate dehydrogenase bypass (PDH bypass), generating acetyl-CoA directly used for lipid or terpene biosynthesis [57]. Additionally, acetyl-CoA hydrolase (EVM0005470, log2 FC = 1.8) can convert acetyl-CoA from other sources into acetate, entering the PDH bypass. Acetyl-CoA serves as a common substrate for the biosynthesis of carotenoids and lipids. Studies have suggested that there is a correlation between elevated carotenoid levels and reduced lipid biosynthetic flux [32]. Our previous data also suggested a positive correlation between fatty acid degradation and carotenoid biosynthesis in *Rhodosporidium kratochvilovae* [58]. This study further reinforced the notions by demonstrating a reduction in lipid content (Figure 3b) resulting from the regulation of key genes expressed that were involved in lipid biosynthesis and the fatty acid β-oxidation. All these results indicated a heightened flow of acetyl-CoA towards carotenoid biosynthesis. (Figure 9). We also found that the expression levels of key genes for carotenoid biosynthesis, specifically phytoene synthase/lycopene β-cyclase (EVM0003148, log2 FC = 0.85) and phytoene desaturase (EVM0006960, log2 FC = 0.59), were upregulated (Figure S1); however, no significant changes were observed in other related genes. Furthermore, this study spontaneously revealed two secondary metabolite genes potentially associated with the regulation of carotenoid biosynthesis: the 4-coumarate--CoA ligase, which plays a role in the terpenoid synthesis pathway and may help combat oxidative stress by triggering ROS clearance [59], and squalene monooxygenase, which promotes the biosynthesis of sesquiterpenes and triterpenes, providing precursors for carotenoids [60]. YM25079 prefers the promotion of high carotenoid biosynthesis rather than using other non-enzymatic antioxidants to counteract ROS stress induced by SB. Moreover, it was also observed that genes related to acetyl-CoA accumulation and carotenoid biosynthesis were co-expressed after treatment with 10 mM. This might be the result of their genomic clustering, as studies have shown that functional clustering of metabolism-related genes appears to be conserved across *Dikarya* [61]. However, further analysis are needed to confirm their correlations after the availability of high-quality genomes of the YM25079.

## 5. Conclusions

In this study, we investigated the effect of sodium butyrate treatment on carotenoid biosynthesis in YM25079. The results indicated that, compared to the control, treatment with 10 mM SB significantly increased carotenoid production. The results of the metabolomic and transcriptomic analyses confirmed that adjustment of energy metabolism, maintenance of membrane integrity and fluidity, enhancement of ROS scavenging capacity, and overall antioxidant activity are the main responses of YM25079 to SB. Carotenoids play a crucial role in scavenging ROS, increasing tolerance to oxidative stress and maintaining the structure and functionality of the cell membrane. Many studies only investigated the induction of carotenoid production by increasing ROS levels through external stimuli such as nutritional stress, osmotic stress, or UV irradiation [62,63]; however, they overlooked the possibility of achieving the same goal by influencing unexplored parameters, such as by inhibiting antioxidant enzyme activity [43]. We elucidated the regulatory mechanism underlying the increase in carotenoid biosynthesis induced by SB, linking epigenetic modifiers with antioxidant enzyme activity. This novel and promising approach may allow for the development of bioprocesses for carotenoid production from yeast. Further ongoing studies aim to identify cheaper and more practical strategies for inducing high oxidative stress levels while ensuring the normal growth and development of yeast. To summarize, this study provided greater insights into the molecular and metabolic basis of YM25079, providing a feasible fermentation process and a molecular foundation for increasing carotenoid production. It also laid the groundwork for the application of epigenetic modifiers in fungal carotenoid production.

## Data Availability

Data are contained within the article.

## Supplementary Materials

The following supporting information can be downloaded at: https://www.mdpi.com/article/10.3390/jof10050320/s1, Figure S1: Functional annotation of DEGs based on gene ontology categorization; Figure S2: RT-PCR verification of differentially expressed genes after addition of 10 mM SB; Figure S3: Classification of metabolites detected in total ion chromatography (TIC); Table S1: Relevant genes and primer sequences verified by transcriptome RT-qPCR; Table S2: Statistical table of sequencing data; Table S3: Statistical table of comparison results; Table S4: Functional annotation of DEGs based on gene ontology in BP, MF and CC; Table S5: KEGG pathway enrichment analysis of metabolome; Table S6: Differentially Expressed Metabolites; Table S7: Metabolic pathways co-enriched with differentially expressed genes and differential metabolites; Table S8: Differentially expressed genes.
