# Supplementary material for: Integrated Transcriptomics and Metabolomics Analysis Reveal the Regulatory Mechanisms Underlying Sodium Butyrate-Induced Carotenoid Biosynthesis in Rhodotorula glutinis
Source: J Fungi (Basel). 2024 Apr 27;10(5):320. doi: 10.3390/jof10050320 (PMC11122558; doi:10.3390/jof10050320)
Supplement: Supplementary file 1 [file jof-10-00320-s001.zip › Supplementary figure.pdf]

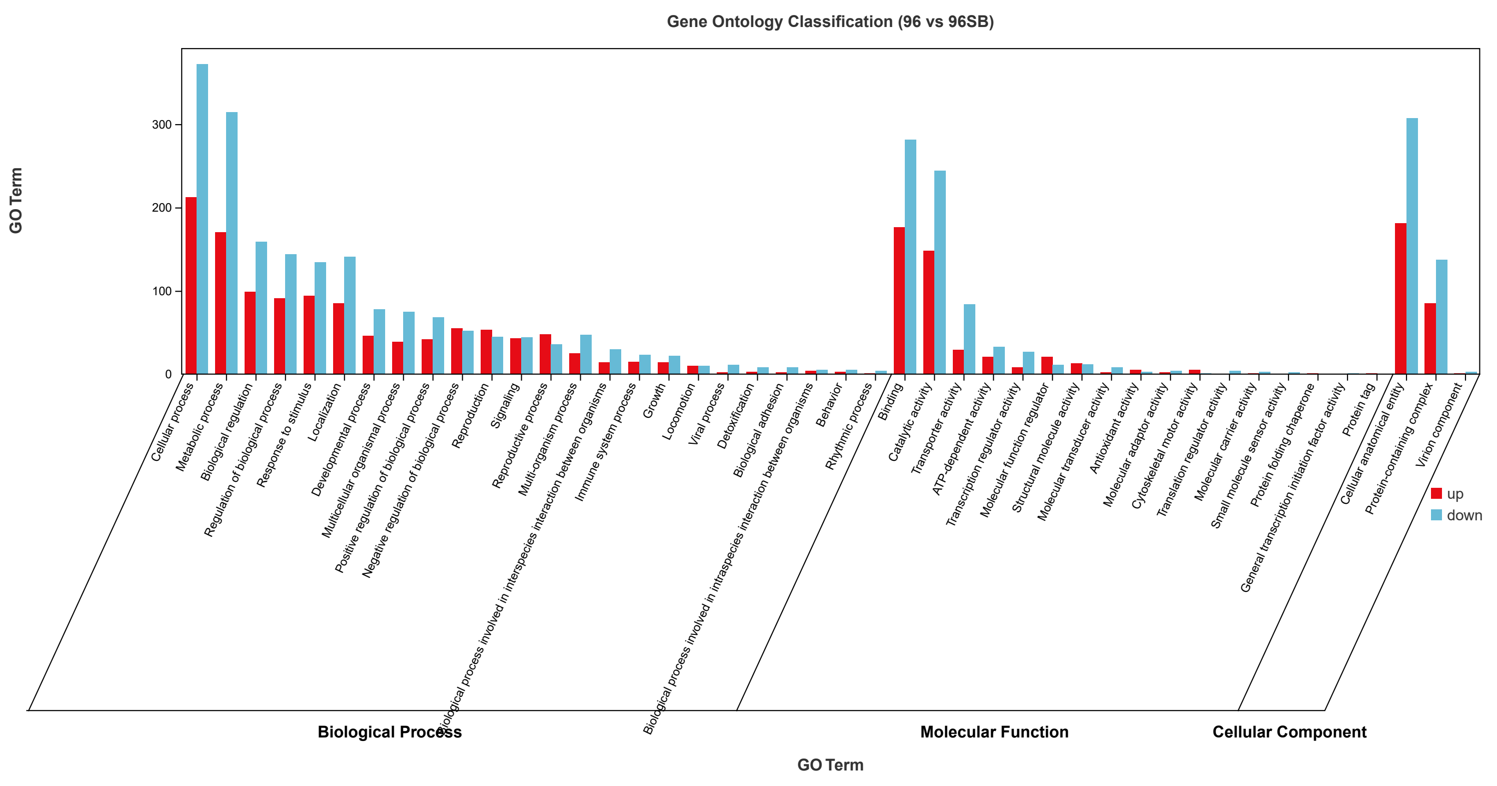

Figure S1. Functional annotation of DEGs based on gene ontology categorization.

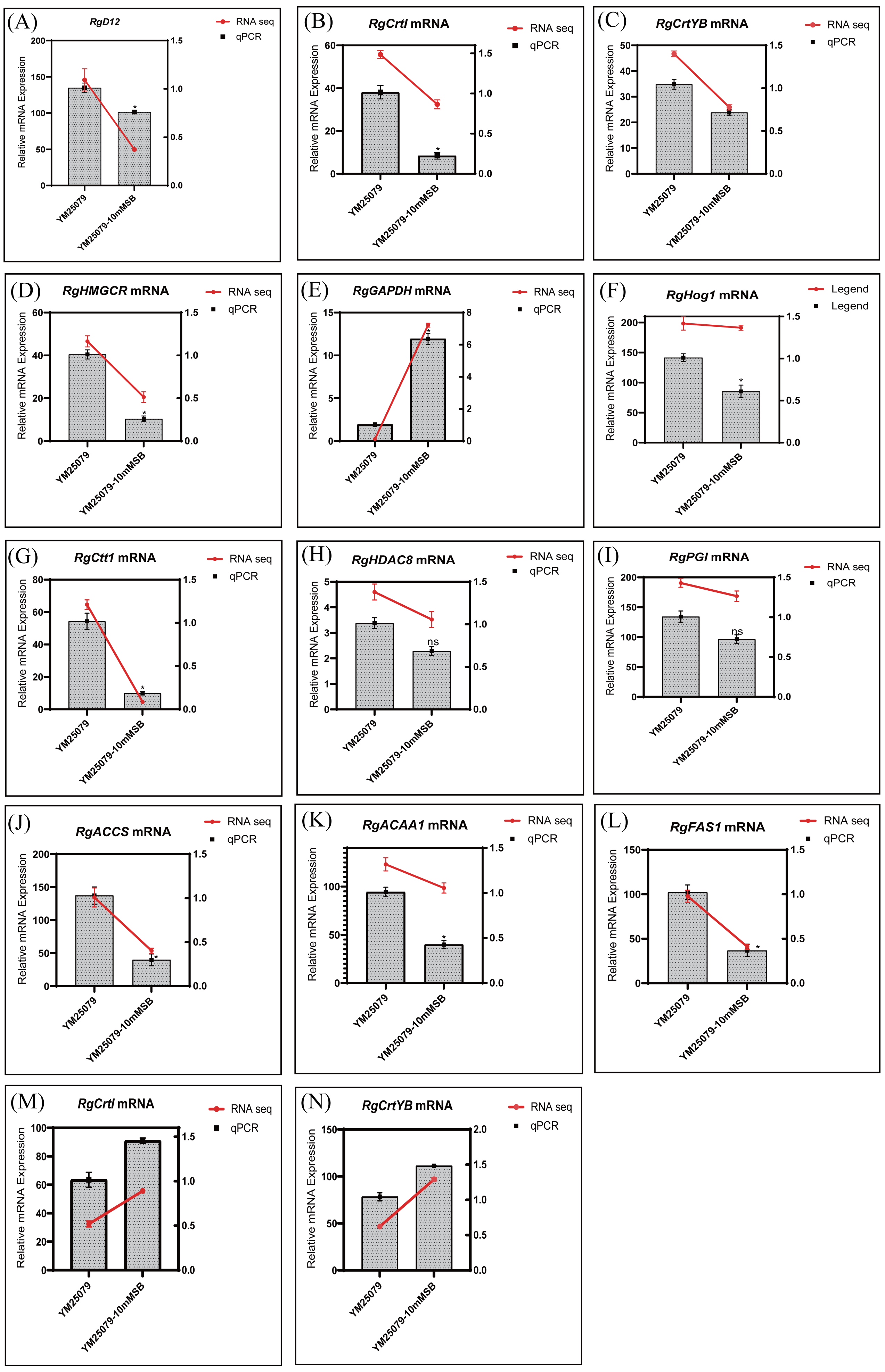

Figure S2. RT-PCR verification of differentially expressed genes after addition of 10mM NaBu.

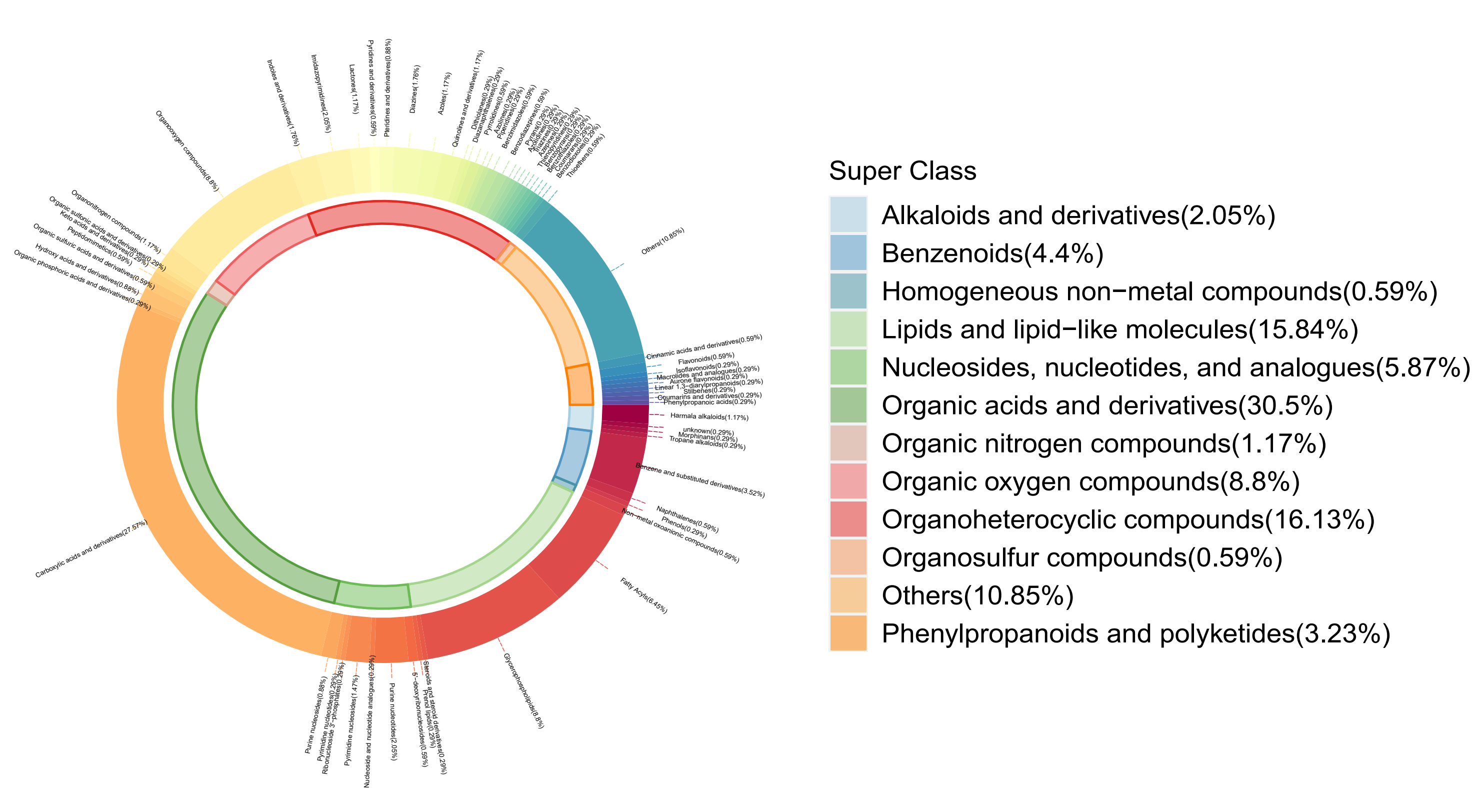

Figure S3. Classification of metabolites detected in total ion chromatography (TIC)
